# A Wireless Sensor System for Real-Time Monitoring and Fault Detection of Motor Arrays

**DOI:** 10.3390/s17030469

**Published:** 2017-02-25

**Authors:** Jonathan Medina-García, Trinidad Sánchez-Rodríguez, Juan Antonio Gómez Galán, Aránzazu Delgado, Fernando Gómez-Bravo, Raúl Jiménez

**Affiliations:** 1 Department of Electronic Engineering, Computers, and Automation, University of Huelva, Ctra Huelva - La Rábida, s/n, 21819 Huelva, Spain; jonathan.medina@diesia.uhu.es (J.M.-G.); trinidad.sanchez@diesia.uhu.es (T.S.-R.); fernando.gomez@diesia.uhu.es (F.G.-B.); naharro@diesia.uhu.es (R.J.); 2 Department of Electrical and Thermal Engineering, University of Huelva, Ctra Huelva - La Rábida, s/n, 21819 Huelva, Spain; aranzazu.delgado@die.uhu.es

**Keywords:** fault detection, IEEE 802.15.4, wireless sensor network, motor current analysis, guaranteed time slot (GTS), beacon-enabled mode

## Abstract

This paper presents a wireless fault detection system for industrial motors that combines vibration, motor current and temperature analysis, thus improving the detection of mechanical faults. The design also considers the time of detection and further possible actions, which are also important for the early detection of possible malfunctions, and thus for avoiding irreversible damage to the motor. The remote motor condition monitoring is implemented through a wireless sensor network (WSN) based on the IEEE 802.15.4 standard. The deployed network uses the beacon-enabled mode to synchronize several sensor nodes with the coordinator node, and the guaranteed time slot mechanism provides data monitoring with a predetermined latency. A graphic user interface offers remote access to motor conditions and real-time monitoring of several parameters. The developed wireless sensor node exhibits very low power consumption since it has been optimized both in terms of hardware and software. The result is a low cost, highly reliable and compact design, achieving a high degree of autonomy of more than two years with just one 3.3 V/2600 mAh battery. Laboratory and field tests confirm the feasibility of the wireless system.

## 1. Introduction

Condition monitoring of industrial motor systems is a high priority task due to their wide use. In developed countries, much of the energy consumed by the industry is due to motor systems; among these, three-phase induction motors are dominant because of their robustness and easy maintenance [1,2,3,4,5,6]. The condition-based maintenance approach uses real-time data, determines the equipment’s health, optimizes maintenance resources and improves the system reliability.

Machine vibration monitoring is one of the oldest and most common approaches due to its high success in preventing failures and the ease of data measurement and analysis. Another method used for fault diagnostic of motor systems is temperature measurement [7]. Excessive friction or bearing deterioration will inevitably cause an increase in the usual motor operating temperature [8]. In addition, high consumption peaks, as well as the loss of a phase in AC induction motors are also parameters to be considered [9,10]. These problems require immediate action to prevent irreparable motor system damage. Practical experience suggests that in order to adequately predict motor system faults it is important to gather as much information as possible. Thus, the combination of the vibration technique with the measurement of other electrical parameters improves the fault detection [11].

This paper presents the design of a wireless system that measures vibrations, temperature and the current consumption of an array of motors in real-time. Real-time monitoring implies that reading of motor data and the subsequent representation are performed periodically, regardless of the additional tasks that the device has to perform [12]. The system allows monitoring the performance with an adjustable sample period using the IEEE 802.15.4 standard in beacon-enabled mode and with a Guaranteed Time Slot (GTS) mechanism, achieving a fixed data monitoring latency. The data from each motor is sent to a base station where decisions about notifying alarms are made. The transducers have been chosen to be compatible with WSN features, i.e., low cost, small size, and low power consumption.

The paper is organized as follows: Section 2 describes the IEEE 802.15.4 standard and the chosen operation mode. Section 3 presents the detailed hardware design for the sensing nodes and coordinator node. Section 4 describes the beacon-enabled mode of the IEEE 802.15.4 standard, the guaranteed time slot (GTS) mechanism, the firmware and the graphical user interface. Section 5 deals with the measurement results. Finally, conclusions are given in Section 6.

## 2. Fundamentals of Wireless Sensor Networks

Wireless systems have increased in popularity, overcoming the complexity of installation, the high maintenance costs and the lack of versatility of the traditional wired systems, especially in remote monitoring. Many wireless technologies have been put to countless different uses in various applications [13].

A wireless sensor network (WSN) consists of wireless sensor nodes or motes, and a sink node or coordinator. The sensors attached to the mote are capable of measuring environmental parameters, and a microcontroller performs data processing. The collected data is transmitted to the coordinator through wireless communication. The radio interface usually incorporates energy-efficient communication techniques to extend the lifetime of the batteries. The coordinator node usually has no constraint on power consumption.

WSNs are included within the category of wireless personal area networks (WPANs). The highly integrated electronics has enabled their rapid expansion by providing small-sized low-power sensor nodes, which facilitate their deployment and ensure high autonomy. Options like 802.11, Bluetooth, UWB, ZigBee, IEEE 802.15.4, etc. are assessed depending on the target application. Within this set of possibilities, in recent years the IEEE 802.15.4 standard has become a benchmark for wireless applications with low data rates and energy efficiency, and will be commonly used in current and future WSN products.

### IEEE 802.15.4: Basic Concepts

This standard, designed especially for Low Rate Wireless Personal Area Networks (LR-WPANs), is a low complexity protocol that provides low cost, low consumption and high versatility to develop various topologies. These features make it an excellent choice for many applications [14,15,16,17,18,19,20]. In addition, the physical layer of communication allows the use of the free frequency industrial-scientific-medical (ISM) band at 2.4 GHz, which is globally available, and thus, a system designed under this standard can be used anywhere in the world.

The deployment of a large number of nodes in the target area, without maintenance for long periods of time, requires power efficiency. The IEEE 802.15.4 standard supports power savings since the nodes can be inactive most of the time, prolonging the lifetime of the network [21].

Although the industry is reluctant to deploy wireless systems because of their low reliability, in the case of the IEEE 802.15.4 standard, security is ensured with the association of the end device nodes to the coordinator nodes of the network.

IEEE 802.15.4 protocol supports two basic modes of operation: non beacon-enabled mode and beacon-enabled mode. For the target application of this work we make use of the beacon-enabled mode since it allows synchronization between the sensing nodes and the coordinator node, and periodic wakeup. Synchronous acquisition is a critical element in the motor vibration monitoring system based on a wireless sensor network. On the other hand, the non-beacon-enabled mode does not support synchronization in the communication and is intended for use in wireless networks that do not require great complexity, i.e., the application can be solved through a sparse network and with little traffic. Thus, the non beacon-enabled mode is useful in networks with a very large number of hierarchical levels, allowing the deployment of networks capable of covering a very large area.

## 3. System Hardware Description

**Figure 1:**
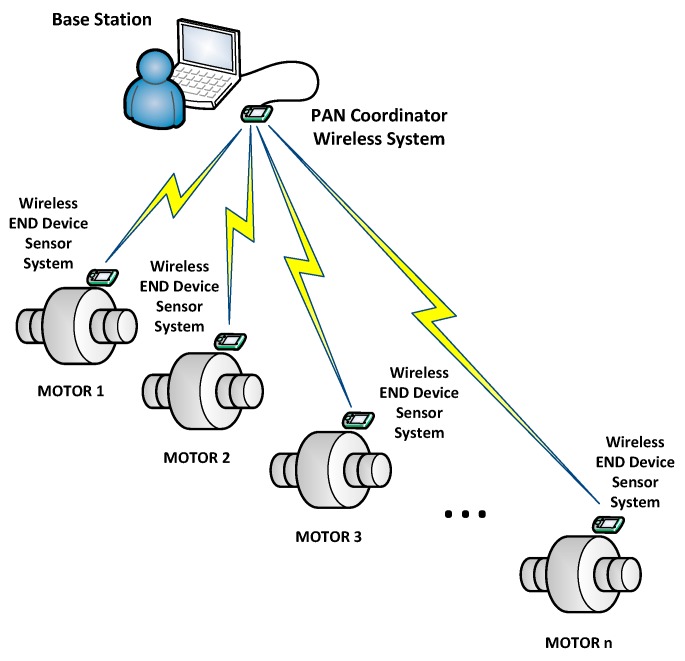
Architecture of the designed wireless sensor network.

This section describes the hardware architecture, the sensors used and the functionality of the system. Figure 1 shows the three different subsystems of the designed system: the sensor devices, the coordinator node and the information management system (base station). The coordinator node is physically connected via USB to the monitoring computer, where data is assessed and processed. The coordinator node gathers all the information generated by the sensor network and transmits it to the monitoring application, which was developed to handle that network. The wireless network has been designed in a star topology. An alternative approach would be to locally process the data extracted from the motors at the sensor level and only transmit the processed results (e.g., fault alarms), reducing the traffic load, but at the cost of increasing the hardware and software requirements of the end sensor devices.

The wireless sensor network system has been developed using devices that meet requirements such as robust radio technology, low cost and reduced size. In addition, low power electronics have been selected for the sensors, conditioning interfaces, microcontroller and transceiver, to ensure appropriate energy handling. The network can be easily scaled up.

### 3.1. Sensor Nodes

**Figure 2:**
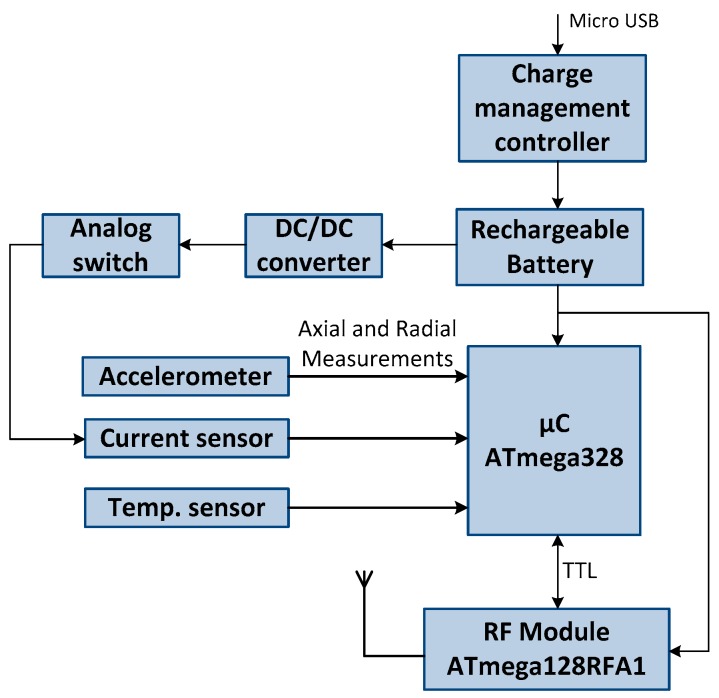
Block diagram of the hardware structure of the sensor nodes.

Figure 2 shows the block diagram of the hardware structure of the sensor nodes. Each mote is structured in a dual-processor architecture to balance cost, power consumption and performance, and consists of two independent circuits: one based on the 8-bit ATmega328 microcontroller from Atmel (San Jose, CA, USA) and another based on the 8-bit ATmega128RFA1 from Atmel. Moreover, it includes a set of three sensors with analog outputs, which measure the required parameters, i.e., vibrations, motor current and temperature. One of the microcontrollers collects the data from the sensors and the other controls the wireless communication stack.

The dual processor design allows for a microcontroller to handle only the communication stack of the IEEE 802.15.4 standard, while the other samples the sensors. If a single microcontroller were used, it would be more difficult to achieve real time monitoring of the vibrations because the number of samples required for an accurate assessment would be more limited. The sensor node is powered by a battery but it can also be operated using an external power supply, via micro-USB.

**Figure 3:**
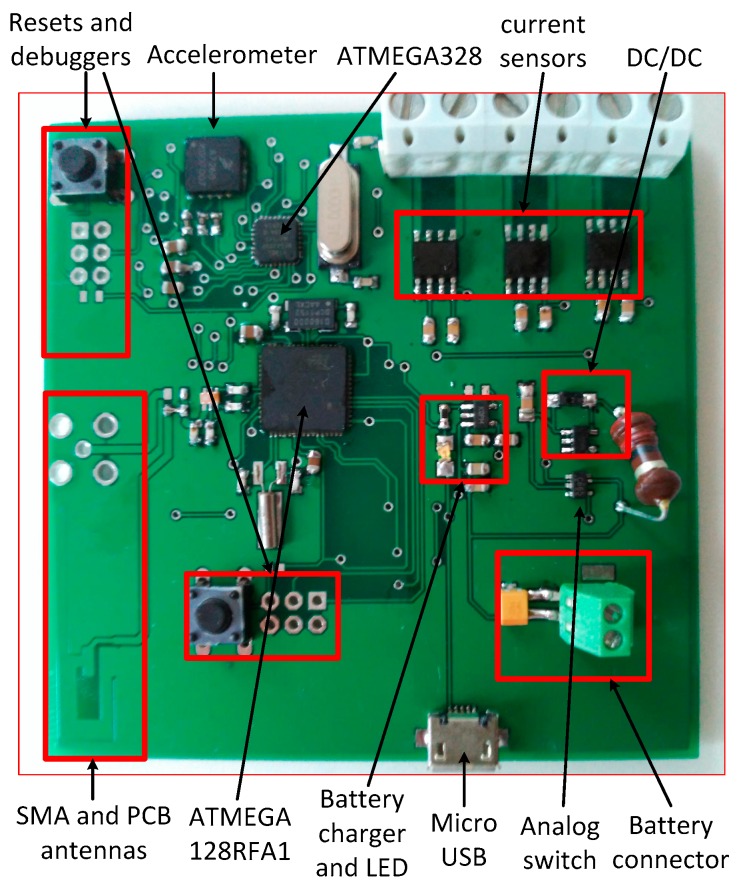
Photograph of the wireless sensor node.

Figure 3 shows details of the hardware implementation of the sensor node.

#### 3.1.1. Wireless Module

The ATmega128RFA1 is a low power 8-bit microcontroller combined with a high data rate transceiver for ZigBee, and IEEE 802.15.4 for the 2.4 GHz ISM band. The radio transceiver provides high data rates, enables very robust wireless communication, and uses a minimum number of external components. It combines excellent RF performance with low cost, small size and low current consumption. For energy optimization, the sensor node will only be powered at specific times for the data gathering process. After the process is completed, it will go into deep sleep mode until the next round of data collection. A low cost solution has been chosen designing a microstrip antenna, avoiding the use of conventional antennas or commercial elements that would add cost to the device.

#### 3.1.2. Microcontroller

Since the ATmega128RFA1 microcontroller is responsible for executing the IEEE 802.15.4 protocol stack, and has to meet the stringent timing requirements of the application, we have chosen to place in parallel another processor to handle the sensors and the communication with the wireless transceiver. This main microcontroller controls and synchronizes the sensor data acquisition process. The output signal of the analog sensors is converted to a binary value using the 10-bit analog to digital converter (ADC) of the microcontroller. The microcontroller transmits the information to the radio transceiver, which handles the physical layer of the wireless communication. Then, the radio transceiver exchanges data with the coordinator node, via wireless at 2.4 GHz.

#### 3.1.3. Accelerometer

The MMA7260QT micro-electro-mechanical systems (MEMS) three axis accelerometer from Freescale (Austin, TX, USA) has been chosen to measure the vibrations. It is a low-cost high sensibility capacitive micromachined accelerometer capable of measuring up to ±6 g in four different sensitivity ranges. The device requires one ADC pin for each axis. This accelerometer has features that make it an ideal solution for the target application, such as reduced package size, and low power and sleep modes with a quick turn on time, which allows the battery life to be extended. In addition, the sensitivity of the device could be easily changed in software.

This accelerometer is based on capacitive sensing cells. Its structure is formed of two back-to-back capacitors and a movable central mass attached to fixed beams, which moves between them. The MEMS accelerometer provides a high voltage level that is proportional to the acceleration. The ADC of the 10-bit microcontroller samples the MMA7260QT and the data sent to the base station is in the form of integer numbers. In the specific case of vibrations, and considering that they are a critical parameter, we have decided to increase the number of samples of each data string. Specifically, the sensing node collects 2048 samples (equivalent to 16,384 bytes) to perform the FFT (Fast Fourier Transform) exactly, yielding 131.072 Kb, which is approximately half the data rate that the IEEE 802.15.4 standard supports. The virtual instrument created in LabVIEW converts the measures of vibrations to millimeters per second (mm/s). Moreover, the internal accelerometer sampling rate of 11 kHz meets the requirements of most applications in machine vibration monitoring, since the most important components in the induction motor vibration spectra is at twice the electrical line frequency [22].

The steps taken to characterize the vibration acquisition are the following: first, the integer data is converted to a decimal value: V*_axial_ = analogread* × 5.00/1024, where *analogread* is the analog integer value collected by the microcontroller and *V_axial_* is the value of the read data in volts. Once calculated the value in volts, the acceleration is calculated in g or m/s^2^: *g_axial_ = V_axial_/Sensitivity*. The sensitivity is set to 0.8 mV/g.

Once the value in “*g*” is known, the sampling rate is set to 33 cycles for a 10 bit conversion, as it is defined in the datasheet of the main microcontroller, together with a cycle frequency of 1 MHz. Thus, the acquisition time is 1 µs, yielding total sampling time for the data acquisition from the four ports of the microcontroller of *t_s_* = 132 µs (1 µs × 33 cycles × 4) [1]. Also, there is a small data transmission time between the two microcontrollers, of a total of 16 µs for the four samples. Therefore, the sampling frequency is set to *f_s_* = 6.756 kHz. Finally, the vibration velocity can be obtained in mm/s as: *vibration = g* × 9.81(1000 × *ts*).

#### 3.1.4. Temperature Sensor

The LM35 (Texas Instruments, Dallas, TX, USA) is a precision IC temperature sensor which output is proportional to the temperature (in °C). The sensor circuitry is sealed and thus, it is not subject to oxidation and other processes. With the LM35, temperature can be measured more accurately than with a thermistor. It also possess low self-heating and does not cause more than 0.1 °C temperature rise in still air. Its operating temperature range is from −55 °C to 150 °C. The output voltage varies by 10 mV in response to every °C rise/fall in ambient temperature, i.e., its scale factor is 0.01 V/°C. The integer data transmitted to the base station from the sensor nodes also requires an almost direct conversion: *temp = analogread* × 100 × 5.0/1024. The sensibility of the temperature sensor is 10 mV/°C, a value addressable by the microcontroller, which is capable of capturing voltage levels below 5 mV.

#### 3.1.5. Current Sensor

In order to monitor the current of the motor, the preferred method of measurement is by means of a Hall Effect-based DC current sensor. The circuit is based on the ACS712 current transducer from Allegro MicroSystems LLC (Worcester, MA, USA). It is a low cost alternative with readily available components and it works well for monitoring the currents encountered in most motor control circuits. This sensor can measure nominal currents from 0 A to 5 A with a sensitivity of 185 mV/A, operating with a supply voltage of 5 V. The current sensor consists of a precise, low offset, linear Hall circuit with a copper conduction path located near the surface of the die. The current flowing through the cooper conduction path generates a magnetic field which the Hall circuit converts into a proportional voltage. The key parameters of the current sensor are summarized in Table 1.

**Figure 4:**
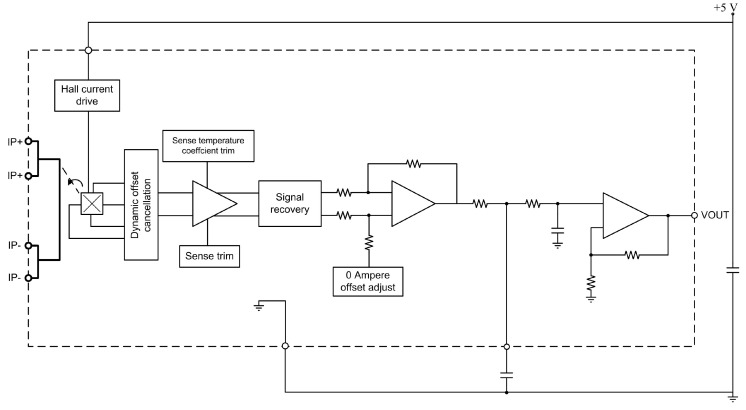
Functional block diagram of the current transducer.

The current transducer uses Hall components to measure the current flowing through the primary winding as shown in Figure 4.

The current in the secondary winding varies linearly with the current in the primary. Finally, an inverting amplifier with single-ended output converts the sensed current into a voltage signal. Again, this output voltage is read by the internal ADC of the microcontroller.

The current transducer has a sensitivity of 0.185 V/A and an offset of 2.5 V. The current of each phase is calculated as: *I_n_ = (analogread − offset)/sensitivity*, where *analogread* is the input voltage at the port of the microcontroller. Since the current sensor does not support the sleep mode, an analog switch has been included to control its shut down, as shown in Figure 2.

#### 3.1.6. Power Consumption

Regarding the sensing node, the study of the power consumption determinates directly the capacity of the battery, and thus, the autonomy. The total power consumption includes the main microcontroller, the radio transceiver and the three sensors, multiplied by the sampling time. The RF transceiver has a power consumption of 250 nA in deep sleep mode and 12.5 mA during data transmission. As each transmission requires 8 bytes, and we intend to send 2048 packets, this yields 16.384 kB. The transmission of 8 bytes of data takes 128 µs, as indicated by the manufacturer. The transceiver power consumption is given by [23]:

$$P_{Node}=\frac{P_{deepsleep}\times T_{deepsleep}+P_{Rx}\times T_{Rx}}{T_{ib}}$$

where *T_ib_* is the mote activity time. This time is calculated by:

$$T_{idle}=T_{tb}^{min}\times Bytes$$

where *T_idle_* is the transmission time set, which is determined by the transmission time of one byte $T_{tb}^{min}$ and the number of bytes to transmit. According to the IEEE 802.15.4 standard, the transmission of eight symbols requires a time of 128 µs. The transmission of data from the sensor node has been set to be hourly. Therefore, as a symbol corresponds to 4 bits, for the transmission of 16.384 kB, it can be estimated that the node is awake 524.288 ms (per hour) for each transmission.

The total power consumption of the sensor node is given by:

$$i_{total}=i_{accelerometer}+\;3i_{ACS712}+i_{ATmega328}+i_{ATmega128RFA1}$$

where *i_accelerometer_* is 1.39 × 10^−7^ mAh, *i_ATmega_*_128*RFA*1_ is 0.0497 mAh, *i_ATmega_*_328_ is 0.08 mAh and 3·*i_ACS_*_712_ is 0.0083 mAh, yielding an average of 0.138 mAh. Since the sensor node battery is 3.3 V and 2600 mAh, its estimated autonomy is 784 days, or just over 2 years.

### 3.2. PAN Coordinator

**Figure 5:**
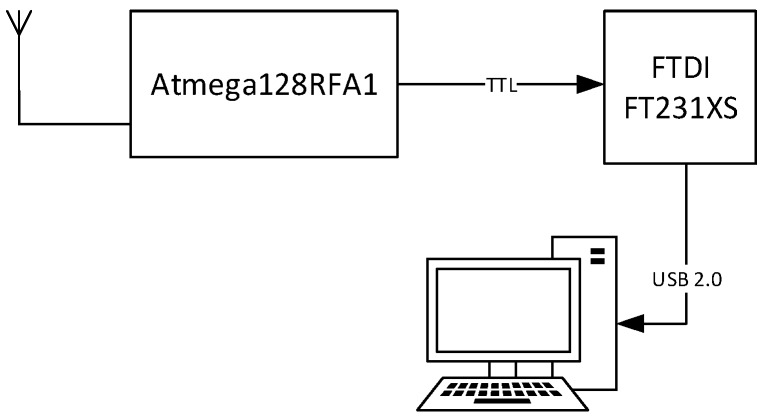
Block diagram of the connectivity of the PAN coordinator.

**Figure 6:**
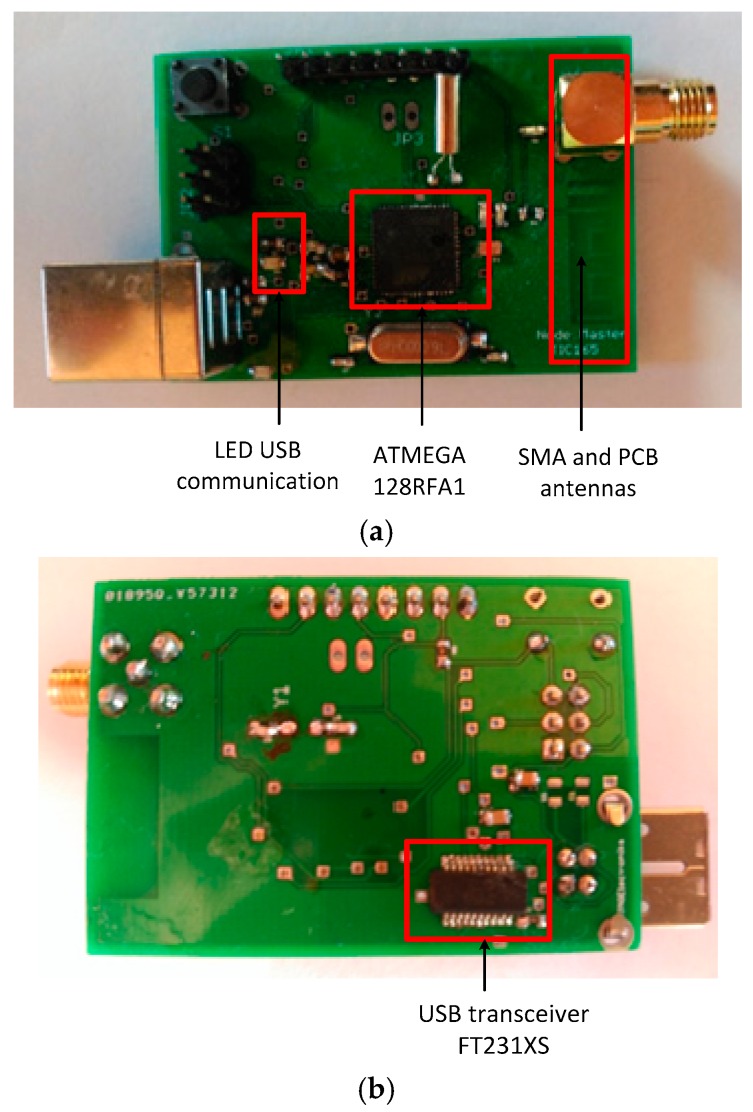
Board of the coordinator node. (**a**) Top; (**b**) Bottom.

The IEEE 802.15.4 standard defines two types of devices: full-function devices (FFD) and reduced-function devices (RFD). In the deployed star network, the sensor nodes or end devices have been set as RFD for their ability to enter sleep mode in which the consumption can be considered negligible. The coordinator node acts as FFD. This kind of node never sleeps, since it needs to relay the information from the sensor nodes to the base station. Therefore, the coordinator also acts as a gateway between the measurement network and the PC, where data is handled by the virtual instrument. The coordinator node is also responsible for managing the network and assigning the addresses to the sensor devices. Figure 5 shows the block diagram of the coordinator node. It includes the 8-bit ATmega128RFA1 microcontroller and the transceiver FT231XS from FTDI. Just like in the sensor node an antenna on the board itself has been implemented. The coordinator node is powered from an external source via USB, and is connected directly to a PC via the TTL-USB transceiver. Figure 6 shows an image of the board of the developed coordinator device.

## 4. Software

**Figure 7:**
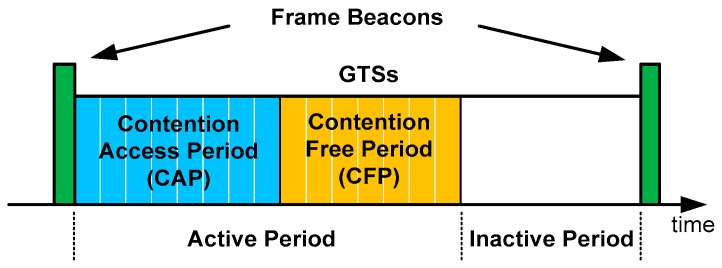
Beacon-enabled mode: superframe structure with GTSs.

The beacon-enabled mode of the IEEE 802.15.4 standard is used since it synchronizes the communication between the coordinator node and the sensor nodes. This approach ensures that the data has been sent, which is essential in this application. The coordinator node periodically sends beacon frames to the associated sensing nodes to synchronize them. The beacon frame is the first part of a superframe, as shown in Figure 7.

The use of this structure allows sensor nodes to track beacons, in order to synchronize their transmissions with the coordinator node. This mechanism allows the nodes media access during the active period, and enables the use of low-power modes during the inactive period of the superframe, in which transmissions are prohibited.

The active period includes two areas: contention access period (CAP) and contention free period (CFP). In the CAP area nodes that want to transmit data to the coordinator node must compete for the media access using the slotted carrier sense multiple access with collision avoidance (CSMA/CA) mechanism. This mechanism provides a media access where each node randomly selects a time slot to transmit, within a range determined by the configurable parameter backoff exponent (BE). A higher value of BE gives a greater range of choice, and therefore a higher average waiting time and a lower probability of collision. Obviously, if the transmission cannot be completed before the end of the CAP area, this will be done in the CAP area of the next superframe. Therefore, it cannot be assured that a transmission can take place within a superframe corresponding to that monitoring instant. To avoid this issue, we make use of the guaranteed time slot (GTS) mechanism to provide data monitoring with a predetermined latency. The coordinator node may allocate up to seven of these GTSs, and a GTS may occupy more than one slot period. Each device transmitting in a GTS ensures that its transaction is completed before the time of the next GTS, or the end of the CFP.

Sensor nodes that transmit within a GTS do not need to use the media access mechanism, because this time slot is reserved for a particular device. Nevertheless, a negotiation protocol must be established between the coordinator node (which sends beacon frames) and the sensing node associated with it. Therefore, the use of GTS allows a monitoring device to transmit information at the same time instant, and at a frequency determined by the periodicity of the superframe.

### 4.1. Handling and Managing of GTS

**Figure 8:**
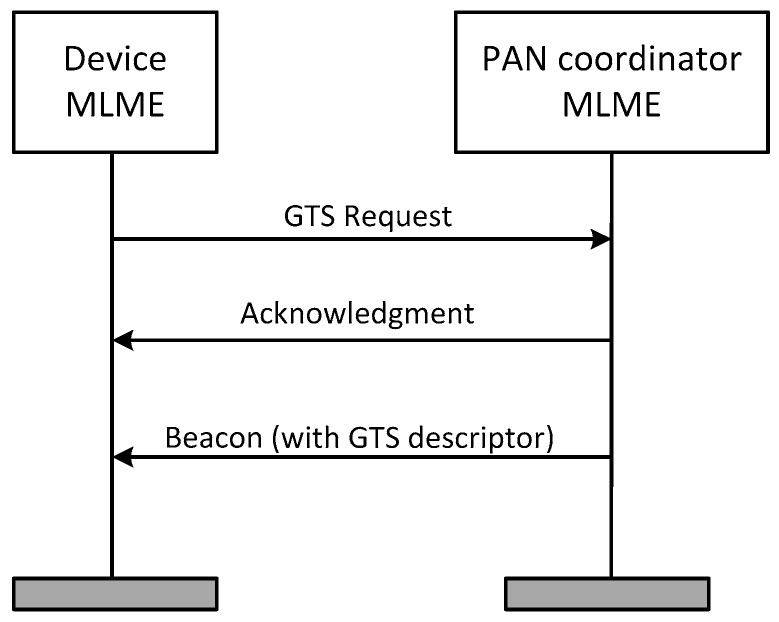
Assignment of GTS to the sensor node.

Each of the nodes responsible for collecting the main motor parameters must reserve a GTS for the transmission of data. Figure 8 shows how the coordinator node assigns a GTS to the sensor node, where MAC Layer Management Entity (MLME) is the MAC management service through which layer management functions may be invoked.

GTS request messages made by the sensor nodes, as well as the acknowledgment to this request by the coordinator node, are transmitted in the CAP area of the superframe, while the affirmative or negative reply to the GTS request is included in the beacon of the next superframe, in form of a GTS descriptor.

**Figure 9:**
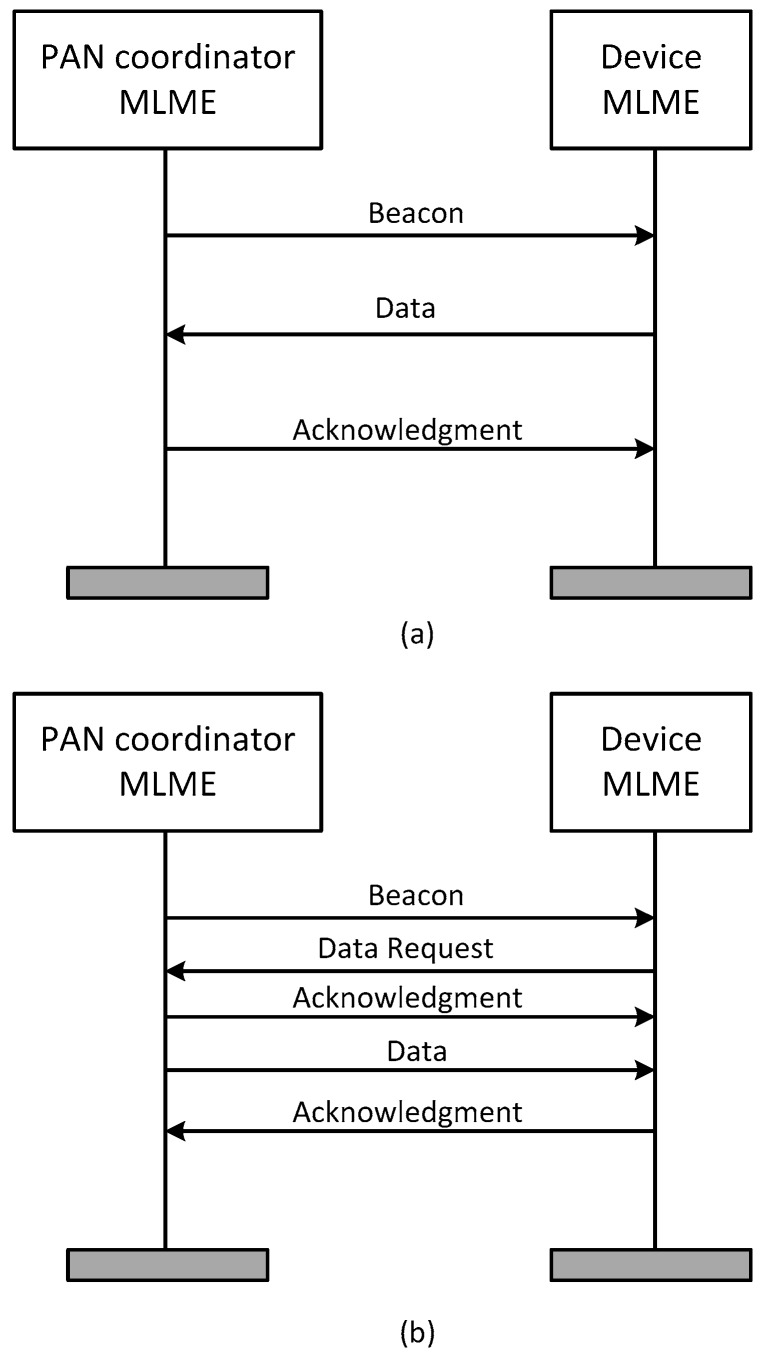
(**a**) Communication from the sensor nodes towards the coordinator node; (**b**) Communication from the coordinator node towards the sensing nodes.

Once the radio link is established and the coordinator has accepted the sensor node within the network, two kinds of communication can be set: an upward one towards the coordinator, and another descending to the sensor node, as shown in Figure 9.

In the first case, shown in Figure 9a, the coordinator node sends a beacon; if the node has data to transmit, it waits until the instant fixed in the GTS, and then performs data transmission and waits for the acknowledgment of the coordinator.

In the case when data is sent from the coordinator node to the sensor node, as shown in Figure 9b, the coordinator sends a beacon. This beacon is followed by a request of data from the sensor node. At this point, the coordinator sends an acknowledgment followed by the data. The transmission ends with an acknowledgment by the sensor node.

### 4.2. Firmware

The modules used for the mechanisms and functionality of the IEEE 802.15.4 standard from the Atmel MAC architecture for IEEE 802.15.4 transceivers are the following:

- Platform Abstraction Layer (PAL): is a module that controls TTL communications, external interrupts, timers and GPIO;
- The MAC module: has the protocol stack of the 802.15.4 communications standard; within this module we must highlight MAC-API, which is the stack for specific applications;
- Resources: includes the management of buffers and the management of Queue;
- TAL: is the transceiver access module, and contains the functions to control the transceiver.

The above modules have been used for both the coordinator node and sensor nodes.

The coordinator’s programming is started by configuring some parameters, such as, Beacon Order (BO), Superframe Order (SO), channel, network name, etc. The parameters BO and SO are defined in the standard, and allow determining the time between beacons and the time in which the end device can transmit, respectively.

**Figure 10:**
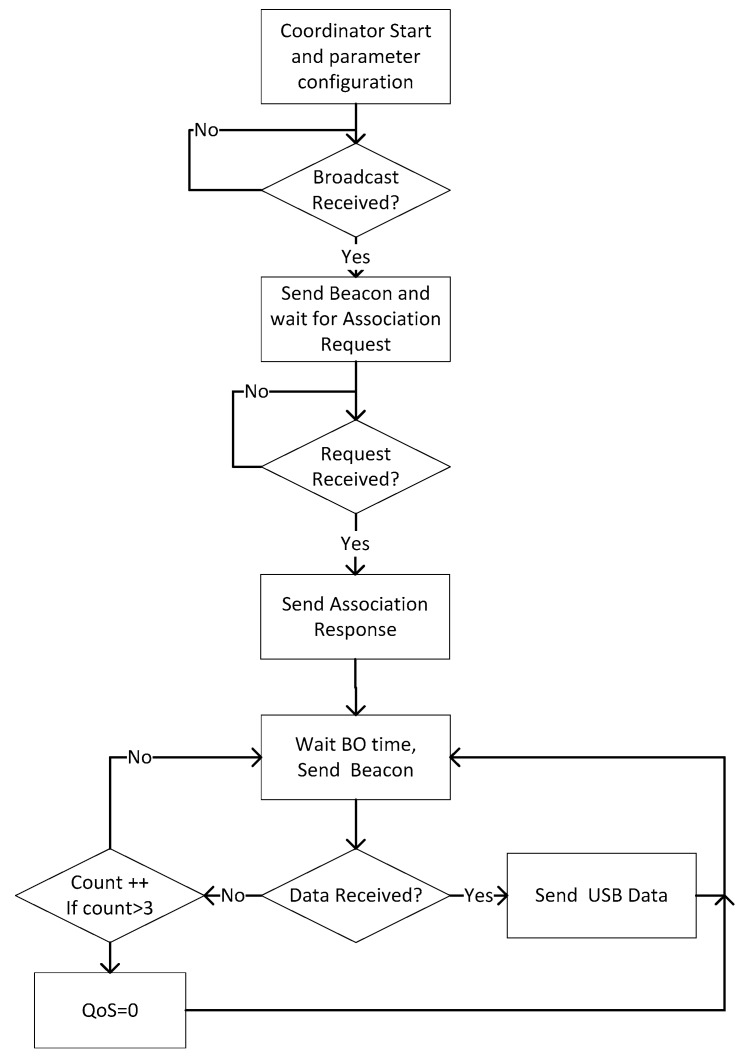
Flow chart of the coordinator node.

Figure 10 shows the flow chart of the coordinator node. Once the parameters are configured so that the coordinating node can create the network, the coordinator waits for some end device (sensor node) to send a beacon. When the coordinator receives the first broadcast (beacon of association sent by an end device), it associates the MAC address of the end device to a motor number, sends an association beacon, and waits for the response of the end device. Once the end device agrees to be associated, it is already within the address buffer of the coordinator. The coordinator waits the time programmed according to the BO and SO parameters before sending the data request beacon. Once this beacon is sent, two events can happen: the coordinator receives the acknowledgement from the end-device and then, since the end device is already associated, the data can be sent to the base station; or, on the contrary, the coordinator does not receive any data, so it sends another beacon. This latter case is programmed in the protocol stack for three attempts, after which the coordinator sends to the base station the data QoS = 0.

**Figure 11:**
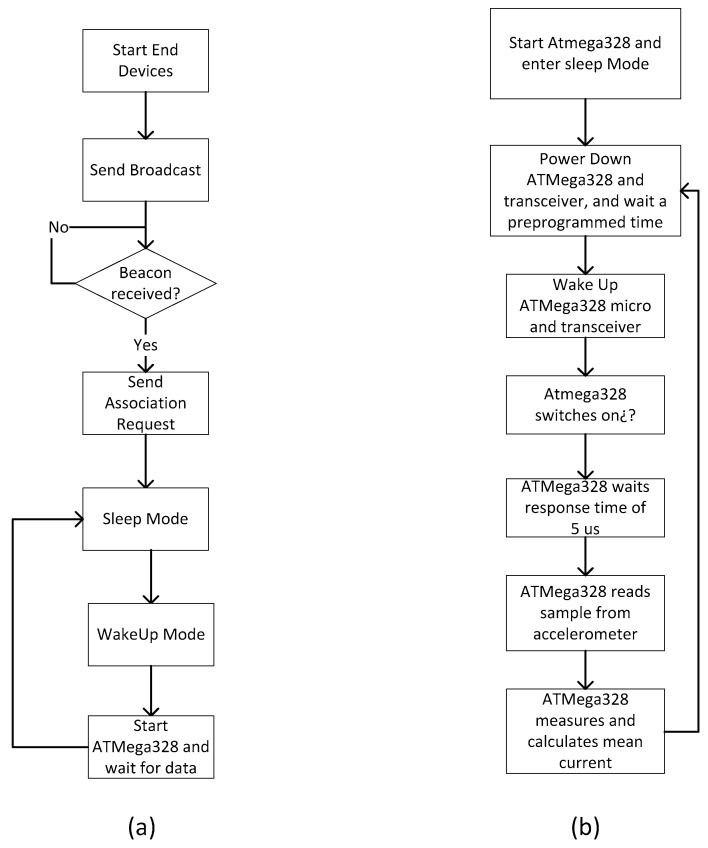
Flow chart of the node sensors. (**a**) Transceiver; (**b**) ATMega328 microcontroller.

As some sensors (the current sensors and temperature sensor) do not have their own low-power programming, they are deactivated and stay that way during the period when they do not take measurements, in order to achieve a low-power strategy. Figure 11 shows the flow chart of the sensor nodes. First, the sensor node makes an association to the network by sending an association frame known as broadcast (within the standard it is defined as association beacon). After the coordinator’s response, the end device accepts the association. Some data is included by the sensor node into the received frame, such as when to wake up to receive a beacon and be able to perform data transmission.

After the specified time, the sensor node wakes up and waits for the arrival of the beacon. The microcontroller also wakes up to manage the sensors. It activates the analog switch (shown in Figure 2) and waits a reasonable time of 5 µs to perform a set of measurements: in particular, it performs 100 current measurements and sends the mean current value to the transceiver. In the case of the temperature it performs exactly the same as with the current measurements. Finally, the microcontroller gathers the measurements of the accelerometers and sends them to the transceiver. At this point the microcontroller enters sleep mode and waits in that state until the transceiver wakes it up again. The transceiver sends to the coordinator node the data to be transmitted to the Virtual Instrument in the base station.

### 4.3. Monitoring Application

**Figure 12:**
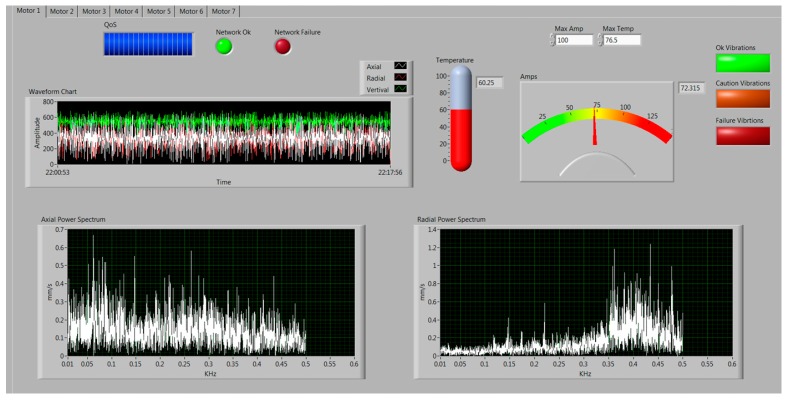
GUI designed for the monitoring application.

Figure 12 shows the monitoring application. The data read by the sensors is sent periodically to the base station.

The graphical user interface (GUI) receives the data and saves it in corresponding database tables according to the data types. The data is processed and analyzed, then graphically visualized in real-time. The received information is relevant for decision making, or for taking appropriate actions. A virtual instrument developed in LabVIEW allows receiving and storing data from the coordinator node. The main features of the graphical interface are summarized below. Several tabs at the top provide the options for each motor system, which in this case, have common functionalities. Each tab includes several configuration settings as well as the limits of the main parameters. Thus, for each of the sensor nodes there can be set the maximum allowable values of temperature, vibration or motor displacement, and current consumption. In the case of exceeding these values, the system triggers an alarm. The GUI also shows the graphical results in the time domain and spectral power in the frequency domain of both axial and radial vibration. In addition, the sampling period can be modified from the user application.

On the order hand, as the data rate from the sensor nodes to the coordinator is 131.072 Kb, 65,536 time slots are needed to perform the transmission correctly. Data of the link-quality indicator (LQI) is also received and monitored by the physical layer. This way it can be seen if the sensing node has lost the radio link with the network coordinator.

## 5. Experimental Results

Several laboratory and field tests have been performed to validate the wireless sensor network. First, laboratory tests have been carried out to verify the reliability of the wireless network, followed by direct measurements from an AC motor, whose results are shown. Next, field tests have been accomplished in a local company.

### 5.1. Reliability of Wireless Communication

A reliability test of the wireless communication is necessary because the proximity of an AC motor generates a noisy environment. To carry out the tests, we used the ceramic antennas integrated into the wireless module, with a gain of 0 dBi.

**Figure 13:**
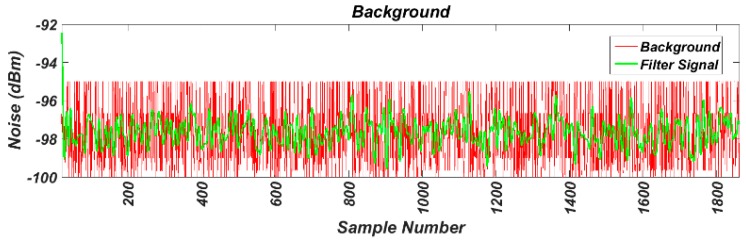
Measured background noise.

**Figure 14:**
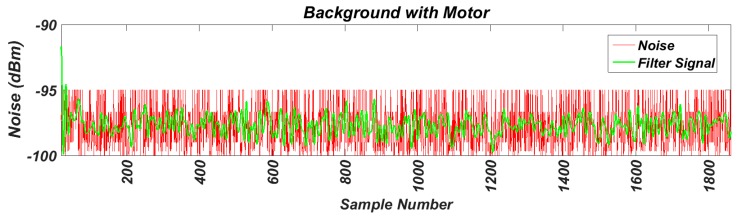
Measured background noise with motors running.

**Figure 15:**
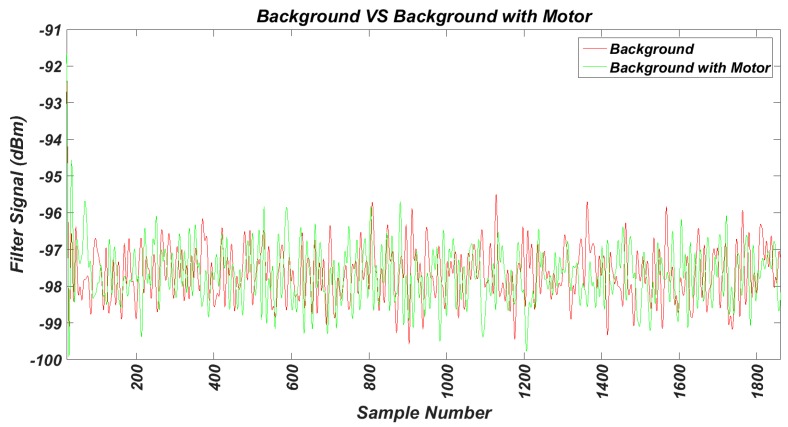
Comparison of the measured and filtered background noise with motors turned off and turned on.

As the first step we have measured the background noise in the band of interest in the work environment, without any motor running. For this test, the transceiver was configured to perform power measurements on 16 channels available in the 2.4 GHz band. The samples were taken at a frequency of 62.5 kHz. The results are shown in Figure 13. Next, using the same transceiver configuration, the background noise has been measured between two running motors, as shown in Figure 14. The comparison of the two noise signals can be observed in Figure 15, where both have been previously filtered.

To assess the results, we calculated the variance and the mean values of the signals, giving the following results:

VarSignal=34.943

MedSignal=−97.2688

VarEngine=35.0387

MedEngine=−97.3068

It can be noted that the operation of the motors does not affect wireless communications.

### 5.2. Laboratory Experimental Setup

**Figure 16:**
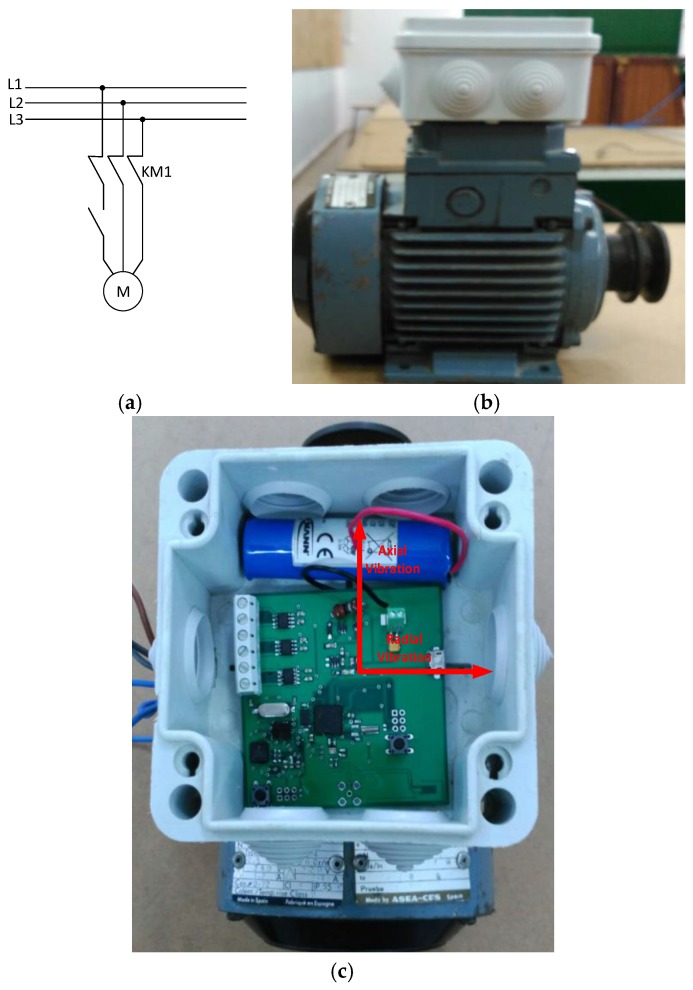
(**a**) Installation diagram; (**b**) Motor and sensor node; (**c**) Detailed photograph of the sensor node embedded into a protection box.

We used a 1LA7063-4AB11 three-phase squirrel cage motor from Siemens [24]. The motor is powered at 400 V with a nominal power of 0.18 kW, and a nominal speed of 1350 r/min. Laboratory tests were performed according to ISO 10816-1 for the Evaluation of Machine Vibration by Measurements on non-rotating parts [25], which classifies the motor system and defines the severity of vibrations. The motor used is Class I (small machine), which states that vibrations exceeding of 7.1 mm/s can damage the equipment. The methodology for testing was as follows: (a) direct start of the motor and sampling of the vibrations performed at 6.756 kHz; (b) subsequent disconnection of one phase of the power system, as shown in the diagram in Figure 16a, causing a breakdown in the motor windings and leading to a considerable increase in motor vibrations. The system generates an alarm when the maximum values are exceeded. Figure 16b shows the motor and the sensor node placement on top of the motor. Figure 16c shows a photograph of the sensor node arrangement inside a protection box that provides shielding against environmental conditions.

In AC motors, vibrations can be caused by several types of faults. A study of the types of faults in motor systems and the natural frequencies that they get to reach is reported in [6]. The vibrations can be axial and radial. Axial vibrations are generated in the flow direction, and radial vibrations occur in the vertical direction to the motor shaft.

**Figure 17:**
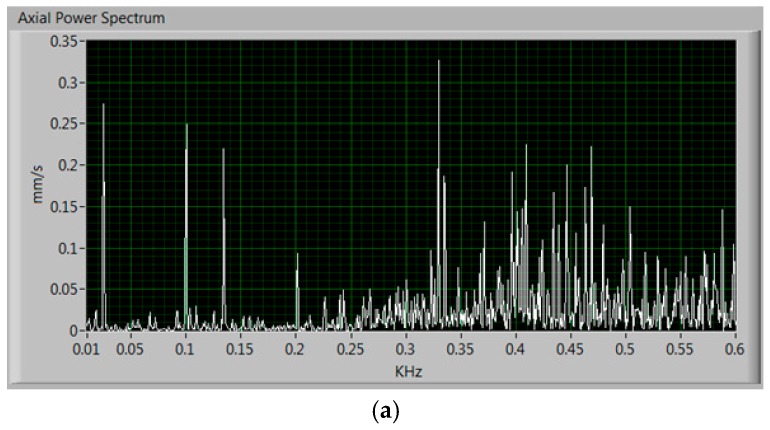

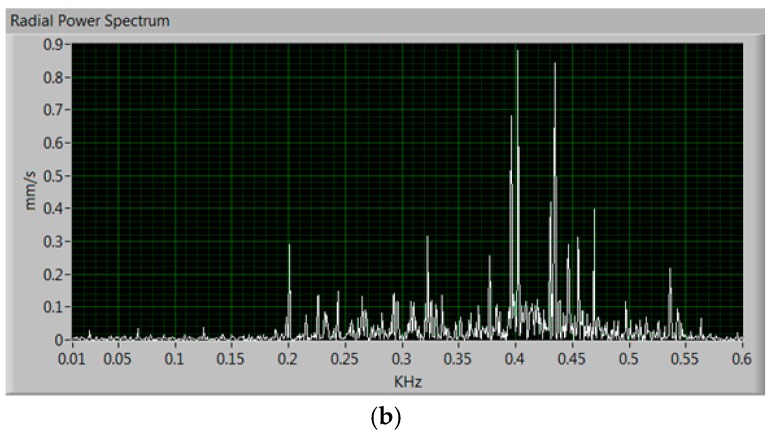
Measured power spectrum of the vibrations when the motor is running properly. (**a**) Axial vibrations and (**b**) radial vibrations.

The overall system feasibility is validated through a set of laboratory experiments. To measure the vibrations, the three-axis accelerometer was employed, of which only two axis were actually used. Figure 17 shows the power spectrum of the measured axial and radial vibrations when the motor is running properly. It can be noted that both values are below 1 mm/s. Although the motor does not present any major failure, it is evidenced that it has some type of wear possibly related to misalignment or a damaged bearing, since it is a motor that is more than 15 years old and that has been submitted to many types of laboratory experiments, some of them very aggressive.

**Figure 18:**
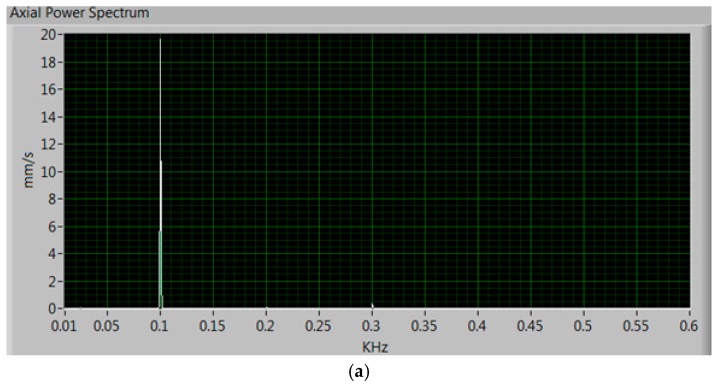

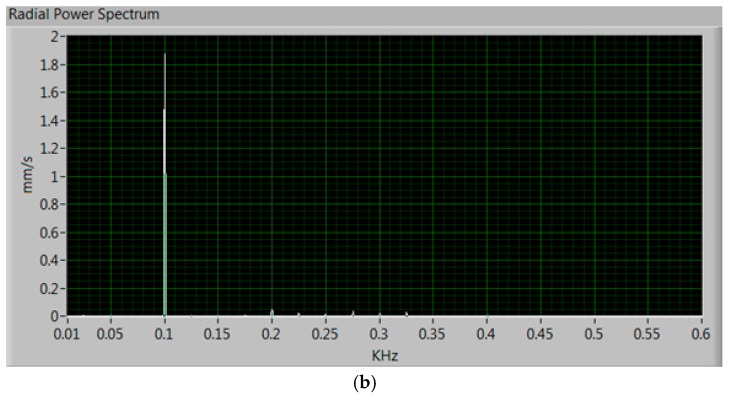
Laboratory measured power spectrum of the vibrations when one phase of the motor is removed. (**a**) Axial vibrations and (**b**) radial vibrations.

Next, a fault to the magnetic flux through a power failure in one phase has been caused [26]. Accordingly, the magnetic flux of the current through the winding leads to an increase of the vibrations in the axial axis as shown in Figure 18. There can be noted the great increase in axial vibrations reaching almost 20 mm/s, which can damage the motor quickly, and thus, it is necessary to stop the motor; the radial vibrations are increased only slightly. Being a motor operating at 50 Hz, according to [22] it has to have a component around 100 Hz, as shown in both figures.

The data length is important for quantifying the power spectrum accurately. Several experimental tests were performed to optimize the number of samples. As it was described in Section 3.1.3 (Accelerometer), 2048 samples were gathered to perform the FFT. This value was chosen as a good tradeoff between power consumption and accuracy. A greater number of samples requires a higher power consumption for their transmission, while a smaller number of samples causes a decrease in the accuracy of the vibration detection. Moreover, a low data rate is also required according to the IEEE 802.15.4 standard used in the target application.

**Figure 19:**
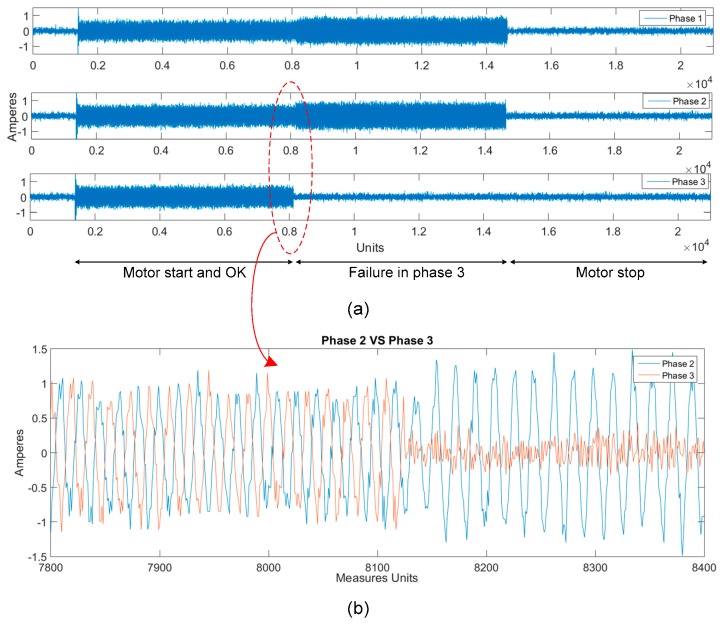
Detection of a fault in a phase by the current sensors. (**a**) Measured current for the three phases; (**b**) Zoom of the currents of the phases 2 and 3 in the area where the phase 3 fails.

Concerning the current sensors they are able to detect a fault in whichever phase as is shown in Figure 19.

Figure 19a shows how at first the motor starts operating properly and it is observed that the current measurement is similar in all three phases. At the time when a failure occurs in one of the phases (phase 3 in the figure), two facts can be noted: the current of that phase is approximately zero, and the current in the other two phases increases significantly. The system is designed to alert of both effects. Figure 19b is a zoom of the zone where the third phase fails, so that the two mentioned effects may be observed with more detail.

### 5.3. Field Tests

**Figure 20:**
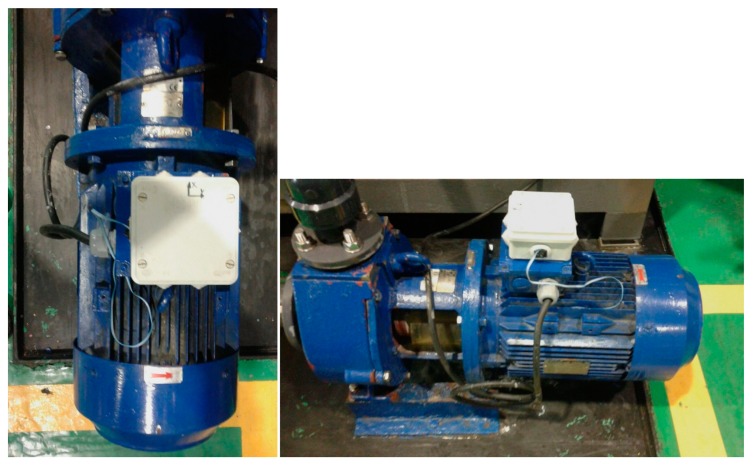
Experimental site for the field test.

In order to test the system in an industrial environment, temperature and vibration tests have been carried out in a local company located in Seville, South-West Spain, with an experience of five decades in the chemical industry. Operators have installed the wireless sensor system in a refrigerated pumping group. Figure 20 shows how the system is placed above the motor housing.

**Figure 21:**
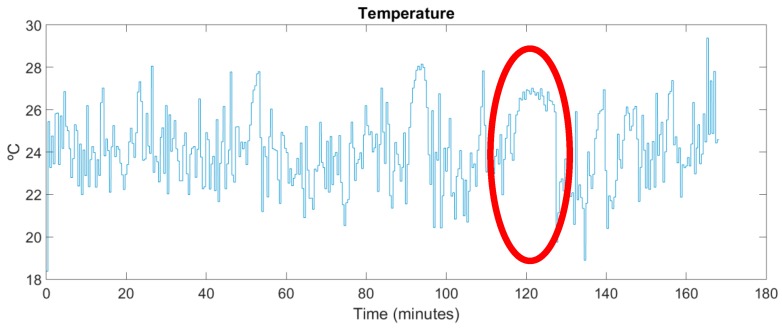
Experimental temperature measurements.

The motor is an 1AR132S2 from WA Motors (Praha, Czech Republic) and the key parameters are: power = 7.5 kW, voltage: 400 V/690 V and a nominal speed of 2915 r/min. When the motor is started to pump liquid in the chemical manufacturing process, a fan is used to cool it down. Figure 21 shows the measurements gathered by the temperature sensor, which is physically located above the motor housing. It can be seen how the motor cooling system acts each time the motor temperature rises.

**Figure 22:**
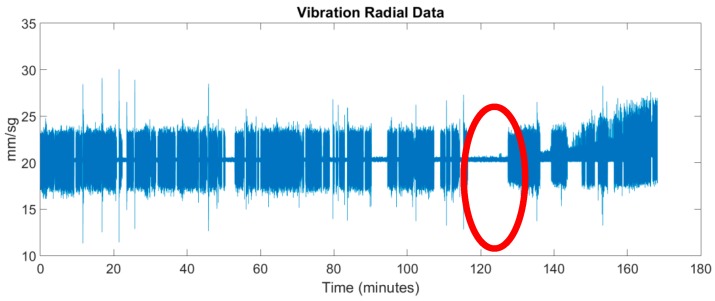
Experimental vibration measurements in time domain.

Figure 22 shows the vibrations measured in real time. Notice how the periods in which the motor is running and the ones when it is stopped are clearly distinguished.

By comparing Figure 21 and Figure 22 it can be verified that when the pumping group starts to operate its temperature decreases, since the cooling system comes into operation. For example, between minutes 117 and 127 the motor is stopped. When it is started again (in minute 127), the cooling system starts to operate lowering the motor temperature. Also note that every time the motor stops, its temperature tends to rise to the ambient temperature of the room where it is located.

**Figure 23:**
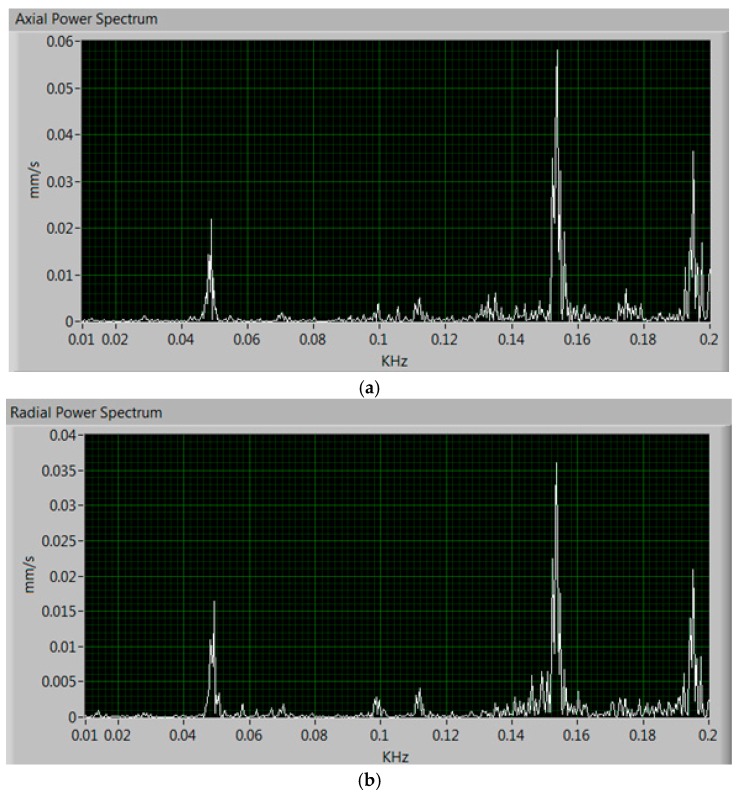
Power spectrum of vibrations measured in the field tests. (**a**) Axial vibrations and (**b**) radial vibrations.

Figure 23 shows the FFT performed on the measurements of the vibrations, in the time domain, and obtained in LabVIEW. It can be noted that the motor does not have any type of mechanical fault.

## 6. Conclusions

This paper presents a wireless sensor network for early detection and monitoring of motor system failures. The system has been designed to merge various parameter measurements in real-time, improving the detectability of faults. The monitoring of the motor system involves the measurement of several quantities, namely vibrations, temperature and current consumption. Thus, compared to a conventional approach that relies solely on vibrations, this design has two more information sources which can trigger an alarm. The wireless communications are based on the IEEE 802.15.4 standard in beacon-enabled mode and with guaranteed time slot. This ensures the data transmission and a synchronous acquisition, which are critical elements in a condition monitoring system based on a wireless sensor network. The data received by the coordinator node is stored and assessed in a PC. The data is graphically presented in real-time by means of a virtual instrument developed in LabVIEW. The proposed system can be easily scaled up to include other sensors on the sensing node for the measurement of other parameters of interest, or to add new sensor nodes to the wireless network. The system has a high autonomy, easy installation and reduced maintenance costs. Experimental measurements confirm the feasibility of the implementation of the sensor network, and its usefulness for the preventive maintenance in three-phase rotating machinery.

## Figures and Tables

**Table 1:** ACS712 current sensor features.

Measuring Range	5 A
Response Time	<5 µs
Power consumption	10 mA
Bandwidth	80 kHz
Sensitivity	185 mV/A
Linearity	1.5%
Accuracy at −40 to 25 °C	0.054 mV/A/°C
Accuracy at 25 to 150 °C	−0.008 mV/A/°C

## References

[1] Lu B., Gungor V.C. (2009). Online and remote motor energy monitoring and fault diagnostics using wireless sensor networks. IEEE Trans. Ind. Electron..

[2] Bogue R. (2013). Sensors for condition monitoring: A review of technologies and applications. Sens. Rev..

[3] Qiao W., Lu D. (2015). Survey on wind turbine condition monitoring and fault diagnosis—Part II: Signals and signal processing methods. IEEE Trans. Ind. Electron..

[4] Lee J., Moon S., Jeong H., Kim W.S. (2015). Robust diagnosis method based on parameter estimation for an interturn short-circuit fault in multipole PMSM under high-speed operation. Sensors.

[5] Peeters C., Guillaume P., Helsen J. (2017). Vibration-based bearing fault detection for operations and maintenance cost reduction in wind energy. Renew. Energy.

[6] Nandi S., Toliyat H.A., Li X. (2005). Condition monitoring and fault diagnosis of electrical motors—A review. IEEE Trans. Energy Convers..

[7] Zaher A.S., McArthur S.D.J. A multi-agent fault detection system for wind turbine defect recognition and diagnosis. Proceedings of the IEEE Lausanne Power Technology.

[8] Guo P., Infield D., Yang X. (2012). Wind turbine generator condition monitoring using temperature trend analysis. IEEE Trans. Sustain. Energy.

[9] Gu F., Wang T., Alwodai A., Tian X., Shao Y., Ball A.D. (2015). A new method of accurate broken rotor bar diagnosis based on modulation signal bispectrum analysis of motor current signals. Mech. Syst. Signal Process..

[10] Yang W., Tavner P.J., Wilkinson M.R. (2009). Condition monitoring and fault diagnosis of a wind turbine synchronous generator drive train. IET Renew. Power Gener..

[11] Ruiz-Cárcel C., Jaramillo V.H., Mba D., Ottewill J.R., Cao Y. (2016). Combination of process and vibration data for improved condition monitoring of industrial systems working under variable operating conditions. Mech. Syst. Signal Process..

[12] Sung W.T., Hsu Y.C. (2011). Designing an industrial real-time measurement and monitoring system based on embedded system and ZigBee. Expert Syst. Appl..

[13] Rawat P., Singh D.K., Chaouchi H., Bonnin J.M. (2014). Wireless sensor networks: A survey on recent developments and potential synergies. J. Supercomput..

[14] Huang Q., Tang B., Deng L. (2015). Development of high synchronous acquisition accuracy wireless sensor network for machine vibration monitoring. Measurement.

[15] Hou L., Bergmann N.W. (2012). Novel industrial wireless sensor networks for machine condition monitoring and fault diagnosis. IEEE Trans. Instrum. Meas..

[16] Sung W.-T. (2010). Multi-sensors data fusion system for wireless sensors networks of factory monitoring via BPN technology. Expert Syst. Appl..

[17] Shariff F., Rahim A.N., Ping W.H. (2015). Zigbee-based data acquisition system for online monitoring of grid-connected photovoltaic system. Expert Syst. Appl..

[18] Sreenithi V., Selvabala N., Ganesh B.A. (2011). Implementation of Wireless sensor network based human fall detection system. Proc. Eng..

[19] Yoo S.-E., Chong P.K., Kim D., Doh Y., Pham M.L., Choi E., Huh J. (2010). Guaranteeing real-time services for industrial wireless sensor networks with IEEE 802.15. IEEE Trans. Ind. Electron..

[20] Salman N., Rasool I., Kemp A.H. Overview of the IEEE 802.15.4 standards family of Low Rate Wireless Personal Area Network. Proceedings of the 7th International Symposium on Wireless Communication Systems (ISWCS).

[21] Li X., Bleakley C.J., Bober W. (2012). Enhanced beacon-enabled mode for improved IEEE 802.15.4 low data rate performance. Wirel. Netw..

[22] Tsypkin M. Induction Motor Condition Monitoring: Vibration analysis technique—A twice line frequency component as a diagnostic tool. Proceedings of the IEEE International Electric Machines & Drives Conference (IEMDC).

[23] Castillo-Effen M., Quintela D.H., Jordan R., Westhoff W., Moreno W. Wireless sensor networks for flash-flood alerting. Proceedings of the Fifth IEEE International Caracas Conference on Devices, Circuits, and Systems.

[24] Siemens Motor. https://mall.industry.siemens.com/mall/en/uk/Catalog/Product/1LA7063-4AB11.

[25] (1995). ISO 10816-1. Evaluation Standard for Vibration Monitoring.

[26] Verucchi C.J., Acosta G.G. (2007). Fault detection and diagnosis techniques in induction electrical machines. IEEE Latin Am. Trans..

